# Preclinical characterization of abemaciclib in hormone receptor positive breast cancer

**DOI:** 10.18632/oncotarget.17778

**Published:** 2017-05-10

**Authors:** Raquel Torres-Guzmán, Bruna Calsina, Ana Hermoso, Carmen Baquero, Beatriz Alvarez, Joaquín Amat, Ann M. McNulty, Xueqian Gong, Karsten Boehnke, Jian Du, Alfonso de Dios, Richard P. Beckmann, Sean Buchanan, María José Lallena

**Affiliations:** ^1^ Quantitative Biology, Eli Lilly and Company, Madrid, Spain; ^2^ Oncology Research, Eli Lilly and Company, Indianapolis, Indiana, USA; ^3^ Discovery Chemistry, Eli Lilly and Company, Indianapolis, Indiana, USA

**Keywords:** abemaciclib, cell cycle, hormone receptor positive breast cancer, senescence, apoptosis

## Abstract

Abemaciclib is an ATP-competitive, reversible kinase inhibitor selective for CDK4 and CDK6 that has shown antitumor activity as a single agent in hormone receptor positive (HR+) metastatic breast cancer in clinical trials. Here, we examined the mechanistic effects of abemaciclib treatment using *in vitro* and *in vivo* breast cancer models. Treatment of estrogen receptor positive (ER+) breast cancer cells with abemaciclib alone led to a decrease in phosphorylation of Rb, arrest at G1, and a decrease in cell proliferation. Moreover, abemaciclib exposure led to durable inhibition of pRb, TopoIIα expression and DNA synthesis, which were maintained after drug removal. Treatment of ER+ breast cancer cells also led to a senescence response as indicated by accumulation of β-galactosidase, formation of senescence-associated heterochromatin foci, and a decrease in FOXM1 positive cells. Continuous exposure to abemaciclib altered breast cancer cell metabolism and induced apoptosis. In a xenograft model of ER+ breast cancer, abemaciclib monotherapy caused regression of tumor growth. Overall these data indicate that abemaciclib is a CDK4 and CDK6 inhibitor that, as a single agent, blocks breast cancer cell progression, and upon longer treatment can lead to sustained antitumor effects through the induction of senescence, apoptosis, and alteration of cellular metabolism.

## INTRODUCTION

Components of the cyclin-dependent kinase 4 (CDK4)/CDK6/Cyclin D/retinoblastoma (Rb)/p16INK4a pathway function in multiple processes implicated in cancer, including cell proliferation, senescence, apoptosis, and cellular metabolism [1–4]. The catalytic proteins CDK4 and CDK6, work with their activating partners, D type cyclins, to phosphorylate the Rb tumor suppressor protein. This phosphorylation of Rb by CDK4/CDK6/Cyclin D, and subsequently by CDK2/Cyclin E, leads to the dissociation of the transcription factor E2F, and release of transcriptional repression of genes important for DNA synthesis and S phase progression [3, 5].

Evidence from breast cancer mouse models suggests that Cyclin D1, and CDK4 and CDK6 activation contribute to suppressing senescence, as Cyclin D1 ablation in these models after tumor formation led to an onset of senescence in the isolated tumor cells, as did treatment with a CDK4 and CDK6 inhibitor [6]. In *KRAS*^G12V^ NSCLC mouse models, conditional knockout of CDK4, but not of CDK2 or CDK6, led to senescence in lung cells, suggesting that certain cell types with particular genetic alterations may be reliant on a specific CDK family member to carry out selected cellular functions [7]. At least one of the downstream mechanisms by which CDK4 and CDK6 suppress senescence is by activating and stabilizing the forkhead transcription factor, FOXM1, through multi-site phosphorylation [8].

Rb also has both pro- apoptotic and anti- apoptotic roles, depending on the cellular context and the balance between Rb activity and the relative activity of other apoptotic or proliferation-related proteins in the cell [1]. The Rb family has also been implicated in glutamine synthesis and mitochondrial function; thus, one additional avenue through which the CDK4/CDK6/Cyclin D/Rb/p16INK4a pathway can control proliferation may be by modulating nutrient availability and the production of cellular energy [2].

The direct contributions of mutations or alterations in this pathway to tumor establishment and/or maintenance are supported by several studies [6, 9–11]. Moreover, evidence suggests that certain tumor types may depend on specific CDK family members for establishment and/or maintenance of tumorigenesis. For example, in mouse models of breast cancers driven by the *HER2/neu* oncogene, loss of CDK4 activity is sufficient to inhibit the formation and proliferation of tumors [10, 12]. Together the available preclinical and emerging clinical data suggest that CDK4 and CDK6 are promising targets for the development of anticancer drugs [13–15].

Abemaciclib (LY2835219) is a small molecule that inhibits CDK4 and CDK6 activity with marked specificity over other CDKs *in vitro* and induces G1 arrest in Rb-proficient cells [16]. In a Phase 1 clinical study (NCT01394016), abemaciclib as single agent had a safety profile which enabled dosing on a continuous schedule, and responses were observed in previously treated patients with HR + metastatic breast cancer (MBC), NSCLC, and melanoma; in addition, confirmation of antitumor activity of abemaciclib as a single agent in HR+ HER2- MBC has been demonstrated in a phase 2 trial [17, 18].

Here we report a mechanistic exploration of the effects of abemaciclib on breast cancer cells. We demonstrate that abemaciclib inhibits Rb phosphorylation and arrests cells in G1 both *in vitro* and in murine models bearing human ER+ breast cancer xenografts. We further show that prolonged and continuous exposure to abemaciclib is accompanied by altered cell metabolism and a substantial increase in markers of senescence and apoptosis in ER+ breast cancer.

## RESULTS

### Abemaciclib is a potent inhibitor of CDK4 and CDK6 that inhibits proliferation of ER+ breast cancer cells

**Figure 1:**
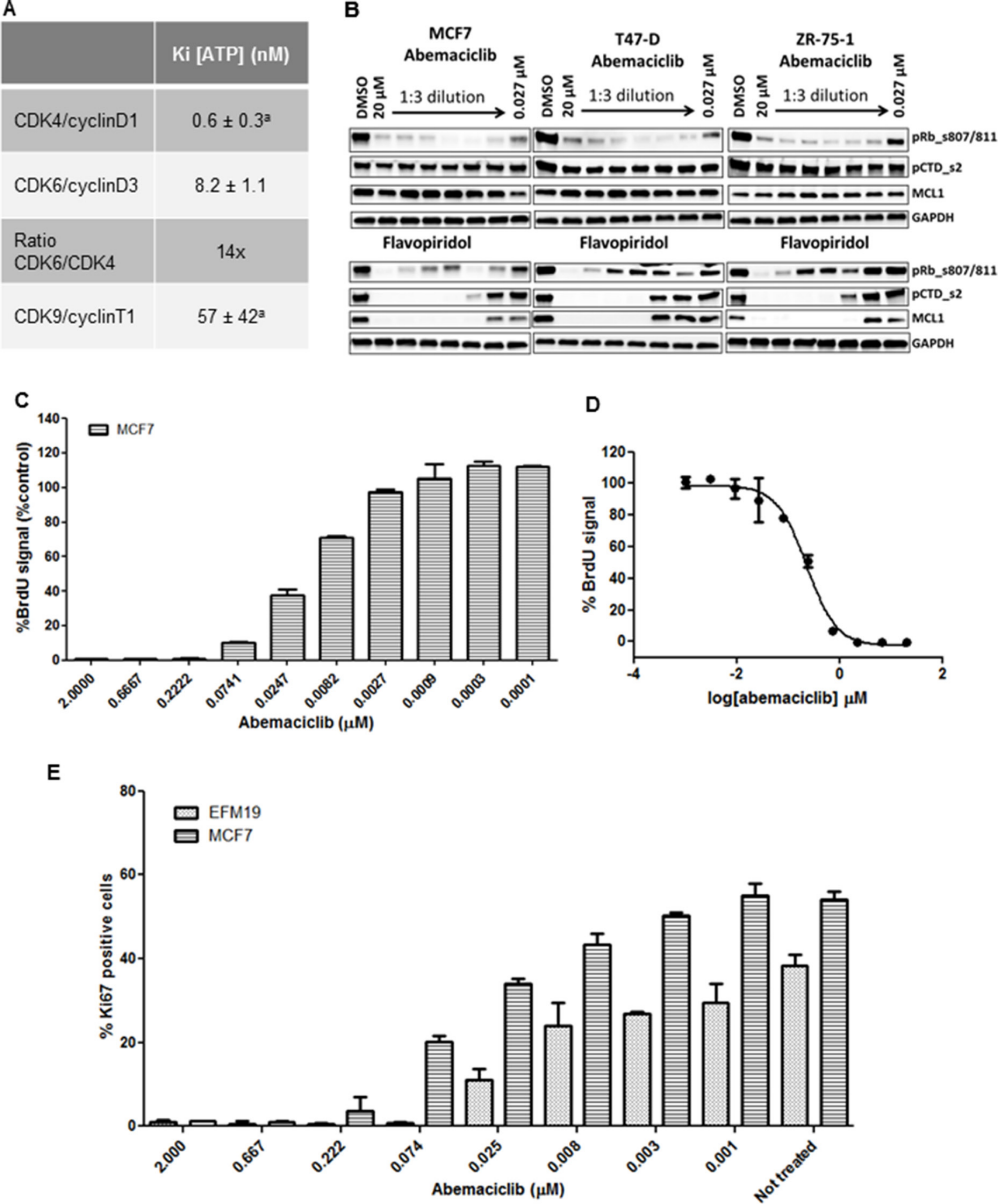
Enzymatic selectivity of abemaciclib and effect on cell proliferation ^a^Values originally published [16]. (**A**) Kinetic parameters (K_i_^ATP^) and selectivity ratio of abemaciclib for cyclinD1/CDK4 and cyclinD3/CDK6 complexes *in vitro*. Data reported as average of two independent determinations (*n =* 2) ± standard deviation (SD) (**B**) ZR-75-1, T-47D and MCF7 cells were treated for 24 hours with DMSO vehicle or with 3-fold dilutions of either abemaciclib or flavopiridol starting at 20 µM and ending at 27 nM. Protein lysates were prepared from the cells and analyzed for the expression of various biomarkers by immunoblotting. The biomarkers included phosphor-Rb at serines 807/811,phosphorylation of serine 2 on the C-terminal repeat domain on RNA polymerase II (pCTD) and MCL1. Images of Western blots were cropped to denote the relevant band(s) for clarity. (**C**, **D**) MCF7 cells were incubated for 3DT with abemaciclib at the concentrations indicated in (C). % BrdU signal was normalized using a PI3K/mTOR inhibitor (BEZ235) as a positive control for inhibiton of cell proliferation and 0.2% DMSO as a negative control (C) and the corresponding IC_50_ curve is shown in (D). (**E**) Ki-67 expression level in MCF7 and EFM-19 cells upon abemaciclib treatment at the indicated concentrations for 2DT. Ki-67 positive events were obtained by gating versus background signal. The percentage of Ki-67 positive cells was calculated among the total number of cells and is expressed as mean (*n =* 2 for samples treated with abemaciclib, *n =* 4 for untreated samples).

Abemaciclib (LY2835219) is an orally available small molecule pyrimidine-benzimidazole inhibitor discovered at Eli Lilly and Company [16]. In biochemical assays, abemaciclib inhibits the kinase activity of CDK4/cyclin D1 complexes with a Ki^ATP^ = 0.6 nmol/L ± 0.3 nmol/L [16] and of CDK6/cyclin D3 complexes with a Ki^ATP^ = 8.2 ± 1.1 nmol/L, indicating that in *in vitro* cell-free assays, abemaciclib shows specificity of approximately 14-fold for CDK4/cyclin D1 over CDK6/cyclin D3 complexes (Figure 1A). Results of enzymatic profiling experiments comparing the inhibitory activity of abemaciclib on several other kinases confirmed its selectivity for CDK4 and CDK6 complexes over other kinases, including CDK9/cyclin T1 complexes (Figure 1A) [16].

To test if abemaciclib also specifically inhibited the CDK4 and CDK6 pathways in cells, we treated MCF7, T-47D, and ZR-75-1 cells for 24 hours with concentrations of abemaciclib ranging from 0.027 µM to 20 µM. Consistent with the mechanism of action of abemaciclib as a CDK4 and CDK6 inhibitor [16], we observed a decrease in levels of phosphorylated Rb (pRb) at S807/811 (Figure 1B). However, even at the highest concentration of abemaciclib, no changes were observed in levels of pCTD_s2 or MCL1, indicating that CDK9-dependent processes were not affected (Figure 1B), consistent with previous observations in U2OS cells [16]. Thus, biochemical and cellular data demonstrate that abemaciclib is a potent and selective inhibitor of CDK4 and CDK6.

Since levels of pRb decreased in ER+ breast cancer lines treated with abemaciclib, and early phase clinical data suggested preferential antitumor activity in patients with HR+ disease [17], we further examined the effects of abemaciclib in ER+ cell lines. A concentration-dependent decrease in viable cell number was observed in ER+ breast cancer cell lines treated with abemaciclib (Supplementary Table 2). Treatment with abemaciclib for 3 DT also led to a concentration-dependent decrease in BrdU incorporation (Figure 1C), with an EC_50_ of 0.178 µM ± 0.0655 in MCF7 cells (Figure 1D). Ki-67 levels decreased in a concentration-dependent manner, in both EFM-19 and MCF7 cells after treatment with abemaciclib for 2 DT, with EC_50_s of 0.0167 µM ± 0.002 and 0.0421 µM ± 0.002, respectively (Figure 1E). These results show that abemaciclib inhibits cellular proliferation in multiple cell lines.

### Abemaciclib inhibited Rb phosphorylation and led to G1/S arrest in ER+ breast cancer cell lines

**Figure 2:**
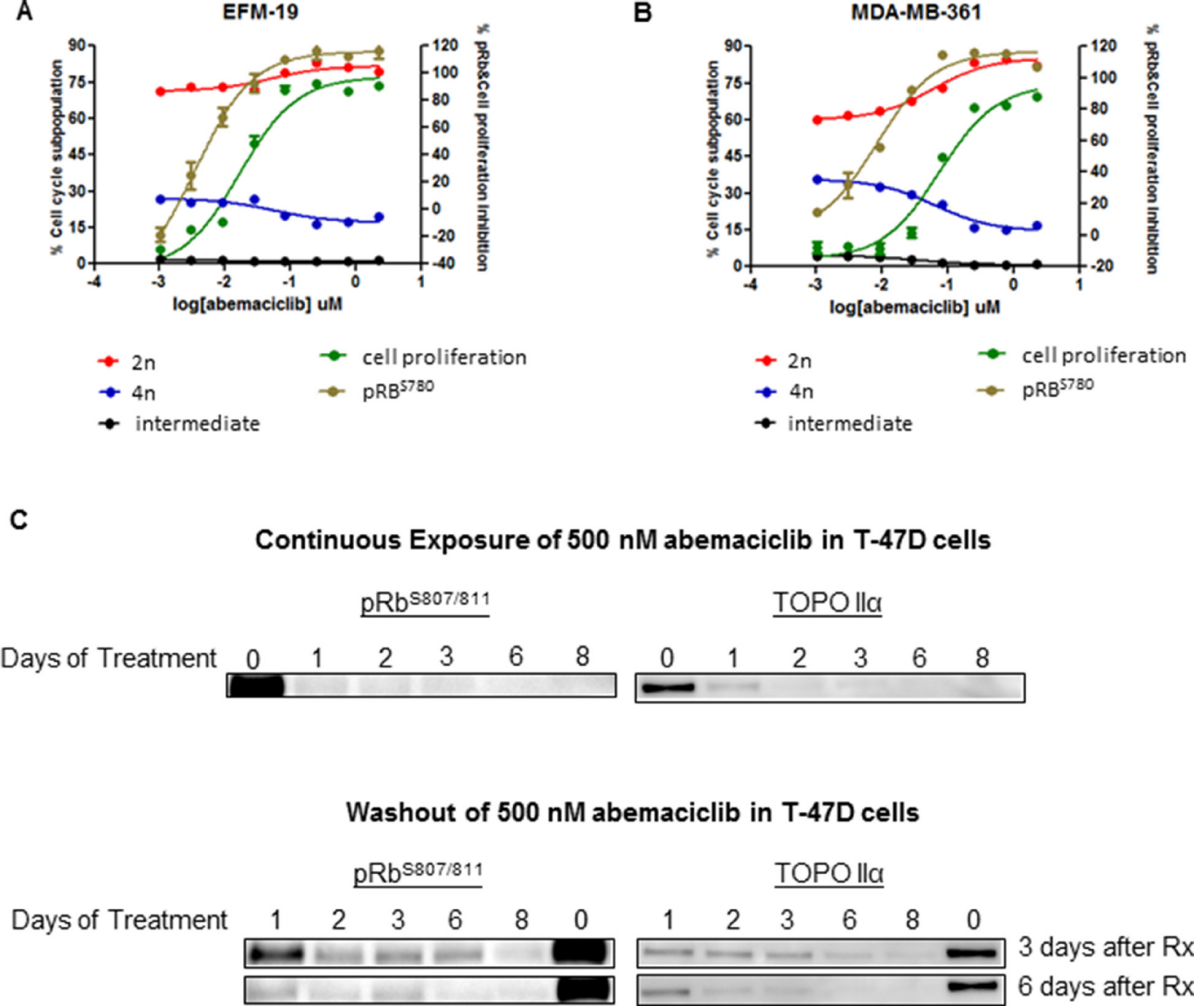
Abemaciclib inhibited cell proliferation, and led to G1/S arrest and a long-term reduction in phosphorylation of Rb in ER+ breast cancer cell lines **(A, B)** Luminal ER+ breast cancer cells were treated with abemaciclib at 8 different concentrations ranging from 2.22 and 0.001 uM. Inhibition of pRb^S780^ (brown line) was measured after 16 hours of treatment by immunofluorescence. Proliferation inhibition (green line) and changes in the cell cycle (red, black, and blue lines) were measured after 4 days (A) and 10 days of treatment (B) by monitoring PI staining. Cell cycle changes are described as the percentage of cells in the 4n subpopulation, (blue), in the 2n subpopulation (red) and in a subpopulation with intermediate DNA content (black). (**C**) T47-D cells failed to reenter the cell cycle following treatment with abemaciclib. T47-D cells were treated for 1-8 days with 500 nM of abemaciclib (continuous treatment) and then were re-plated at 30–40% confluency and maintained in fresh growth medium without abemaciclib for 3 or 6 additional days (washout). Protein lysates were prepared from the cells and the inhibition of cell cycle reentry was measured by monitoring the inhibition of pRb (S807/S811) and Topo IIα expression by immunoblotting. Images of Western blots were cropped to denote the relevant band(s) for clarity.

The effects of abemaciclib on Rb phosphorylation and the cell cycle were assessed *in vitro* in the luminal ER+ breast cancer cell lines EFM-19 and MDA-MB-361. Treatment of these cell lines with abemaciclib for 16 hours, led to a decrease in Rb phosphorylation at Ser 780, in a concentration-dependent manner (Figure 2A, 2B; Supplementary Table 2). Moreover, treatment of EFM-19 and MDA-MB-361 cells with abemaciclib for 2 DT led to an increase in the percentage of cells in the 2n subpopulation and a corresponding decrease in the percentage of cells in the 4n subpopulation, which correlated with the concentration-dependent inhibition of pRb (Figure 2A, 2B; Supplementary Table 2). Together, these results demonstrate that abemaciclib is a potent inhibitor of CDK4 and CDK6 that effectively inhibits the phosphorylation of Rb, leading to cell cycle arrest in G1 with a corresponding decrease in cell proliferation.

Moreover, the observed decreases in pRb and cell cycle progression, as monitored by levels of TopoIIα, were sustainable with continuous exposure of breast cancer cells (T-47D) to abemaciclib, with increased effects observed with longer initial treatment times of abemaciclib (Figure 2C). DNA synthesis, as measured by EdU incorporation, was preferentially reduced in HR+ compared to HR- cell lines, and this inhibition was sustained even after short-term treatment with abemaciclib (Supplementary Figure 1B–1D). HR- cell lines with RB1 mutation or loss including MDA-MB-468, BT549, BT20, HCC1937, HCC70 [21–23] were insensitive to these effects of abemaciclib (Supplementary Figure 1B–1D). Palbociclib treatment also led to a decrease in proliferation preferentially for HR+ cell lines; however, this effect was only observed after longer treatment times (Supplementary Figure 1B–1D). Those data suggest that long term treatment with abemaciclib could lead to sustained response measured by pathway engagement (pRB, Topo IIα) and cell proliferation inhibition. Further experiments should be done to investigate the failure of cells to entry in cell cycle and re-gain proliferative ability. Abemaciclib induced senescence in ER+ breast cancer cell lines

**Figure 3:**
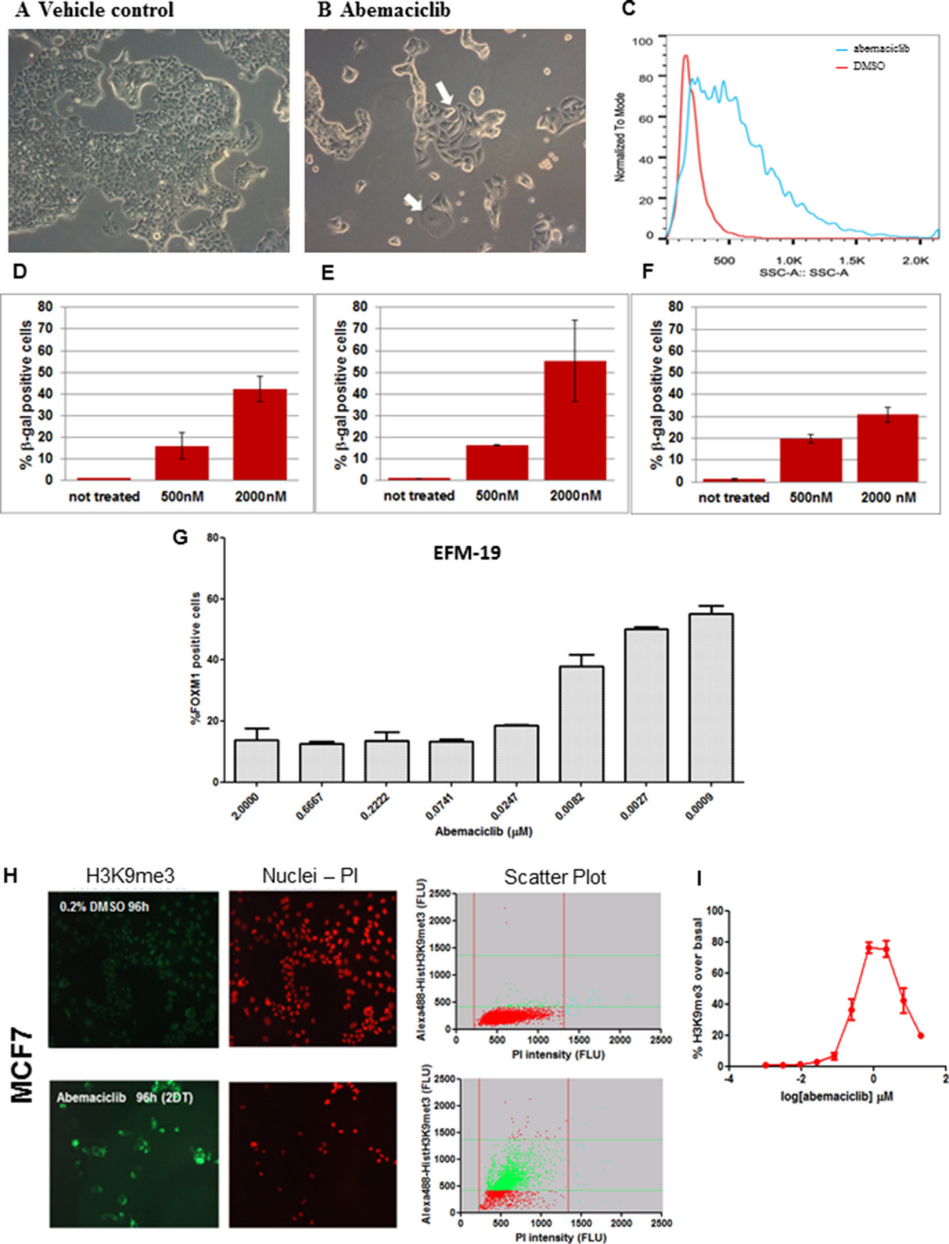
ER+ breast cancer cell lines treated with abemaciclib showed morphological changes, increased β-gal expression and chromatin changes consistent with senescence **(A, B)** MCF7 cells were treated with DMSO 0.2% (A) or 0.5 µM abemaciclib (B) for 3DT and images were taken at 100x by bright field microscopy (Leica DMiL). White arrows indicate evident morphological changes enlarged and flatten cytoplasm upon treatment with abemaciclib. (**C**) MCF7 cells were treated with either DMSO (red line) or 0.5 µM of abemaciclib (blue line) for 3 DT and side scatter (SSC) analysis was carried out using flow cytometry and analyzed with FlowJo X software. (**D**–**F**) MCF7 cells were treated with abemaciclib for 2 days (D), 7days (E), or 10 days (F) and β-gal was measured by high-content imaging (HCI). For abemaciclib treated samples (n =2), and for untreated cells (*n =* 6), the means (SD) are plotted. (**G**) EFM-19 cells were treated for 1DT with abemaciclib at the indicated concentrations. FOXM1 positive cells were detected by HCI; signal for % FOXM1-positive cells is expressed as mean (SD) (*n =* 2 for abemaciclib treated samples and *n =* 4 for untreated samples). (**H**) MCF7 cells were treated with DMSO or 0.5 µM abemaciclib for 2DT and immunofluorescence was carried out with an H3K9me3 antibody and PI. Microscopy images are shown on the left and scatter plots of total and H3K9me3 positive cell populations are represented on the right (Red = nuclei; green = H3K9me3 positive cells). (**I**) MCF7 cells were treated for 2 DT with different concentrations of abemaciclib. The % of H3K9me3 positive cells was obtained by normalizing versus a control population and expressed as the number of objects stained with H3K9me3 antibody; the mean (SD) of 2 independent experiments at each concentration is plotted.

Since the CDK4/CDK6/CyclinD/Rb/p16INK4a pathway plays a role in preventing senescence, we explored whether treatment with abemaciclib could induce senescence. We observed that MCF7, MDA-MB-361 and EFM-19 cells treated for 3 DT with 0.5 µM abemaciclib were larger and flatter compared to vehicle-treated cells in all three cell lines (Figure 3A, 3B and Supplementary Figure 2A–2D). Moreover, morphological characteristics, as assessed by side scatter measurements using flow cytometry, indicated an increase in overall cellular complexity, consistent with a senescent phenotype (Figure 3C and Supplementary Figure 2E, 2F).

Increased β-gal staining was also detected in MCF7 cells treated with 0.5 µM or 2 µM abemaciclib for 4,7 and 10 days, compared to cells treated with DMSO (Figure 3D–3F, Supplementary Figure 2G, 2H). Overall, the percentage of cells with detectable β-gal increased with higher concentrations of abemaciclib such that at 2 µM, 55.4 +/–18.8% cells were β-gal positive (Supplementary Table 3). Notably, the increase in β-gal staining was apparent even after treatment duration as short as 4 hours with concentrations similar to those observed in plasma in humans (data not shown). Longer incubation times generally led to an increase in the percentage of β-gal positive cells observed, although at the highest concentration (2 µM) and longest incubation time (10 days), a decrease was observed (Figure 3D–3F, Supplementary Table 3), along with a concomitant reduction in viable cell number (data not shown).

Consistent with cells having entered a senescent state, a concentration-dependent reduction in FOXM1 positive EFM-19 cells (IC_50_=0.008 µM ± 0.005) was observed after 1 DT (Figure 3G). These data are consistent with observations that a decrease in FOXM1 levels due to CDK4 and CDK6 inhibition is involved in promoting senescence [8]. We further assessed senescence-related chromatin changes in cells treated with abemaciclib, and observed higher levels of trimethylation of histone H3 at lysine 9 (H3K9me3) in MCF7 and EFM-19 cells after 2 DT of treatment with 500 nM abemaciclib compared to cells treated with 0.2% DMSO (vehicle), consistent with formation of senescence-associated heterochromatin foci (SAHF) (Figures 3H and Supplementary Figure 2I, Supplementary Table 4). This staining showed that vacuolization was increased during treatment with abemaciclib, demonstrating an additional feature of senescence. PI staining also indicated fewer viable cells in the samples treated with abemaciclib, again indicating an impact on cell viability (Figures 3H and Supplementary Figure 2I). The increase in H3K9me3 in cells treated with abemaciclib was concentration-dependent up to 2 µM, achieving maximum levels in MCF7 cells at clinically-achievable concentrations (Figure 3I, Supplementary Figure 2J). In EFM-19 cells, abemaciclib led to increased expression of H3K9me3 up to 20 µM for this marker with a potency of 0.023 µM (Supplementary Figure 2J and Supplementary Table 4). These observations indicate that abemaciclib induces senescence, as signified by characteristic morphological changes, expression of β-gal, and increases in H3K9me3.

### Programmed cell death in breast cancer cell lines treated with abemaciclib

**Figure 4:**
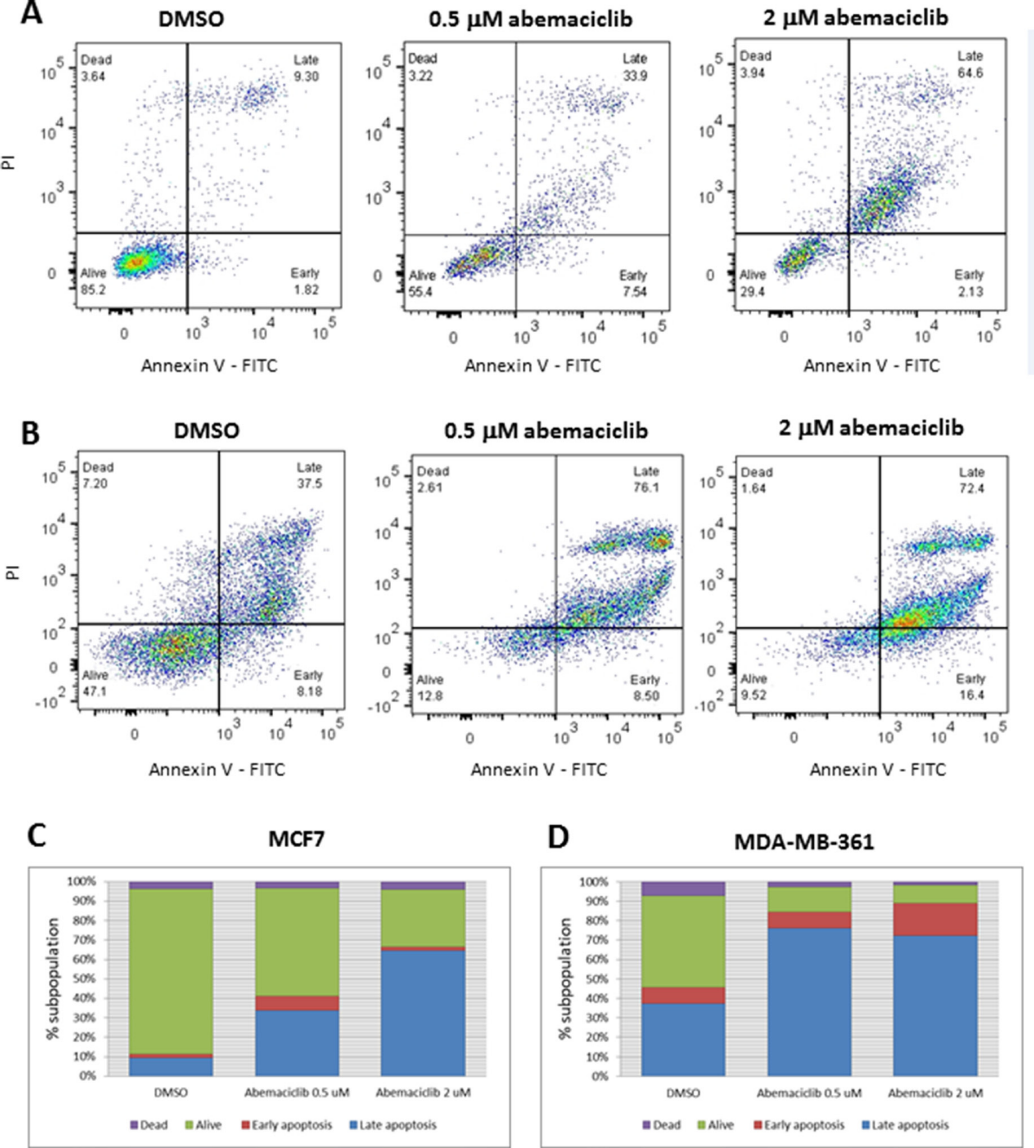
Apoptosis in breast cancer cell lines after abemaciclib treatment, as measured by annexin V binding **(A, B)** MCF7 (A) and MDA-MB-361 (B) cells were treated with the indicated concentrations of abemaciclib for 3 DT or treated with 0.2% DMSO. Flow cytometry plots for annexin V (X-axis)/propidium Iodide (Y-axis) staining are shown. (**C**, **D**) Bar plots showing percentages of annexin V and of PI, representative of early apoptosis, late apoptosis, live and dead cells in MCF7 (C) and MDA-MB-361 (D). Each experiment was performed in independent replicate experiments and a representative example is shown.

The observations described above suggest that as the concentration and duration of abemaciclib treatment increased, an increase in cell death was occurring. Consistent with this observation, dose-dependent increases in annexin V staining were observed in MCF7 and MDA-MB-361 cells treated with abemaciclib for 3 DT (Figure 4). In untreated MCF7 cells, 11.1% of cells were positive for annexin V, whereas 41.4% and 66.7% of cells were positive for annexin V when treated with 0.5 µM and 2 µM of abemaciclib, respectively (Figure 4A, 4C). Likewise, in untreated MDA-MB-361 cells, 45.7% were annexin V-positive, compared with 84.6% and 88.8% upon 3 DT of treatment with 0.5 µM and 2 µM abemaciclib, respectively (Figure 4B, 4D).

**Figure 5:**
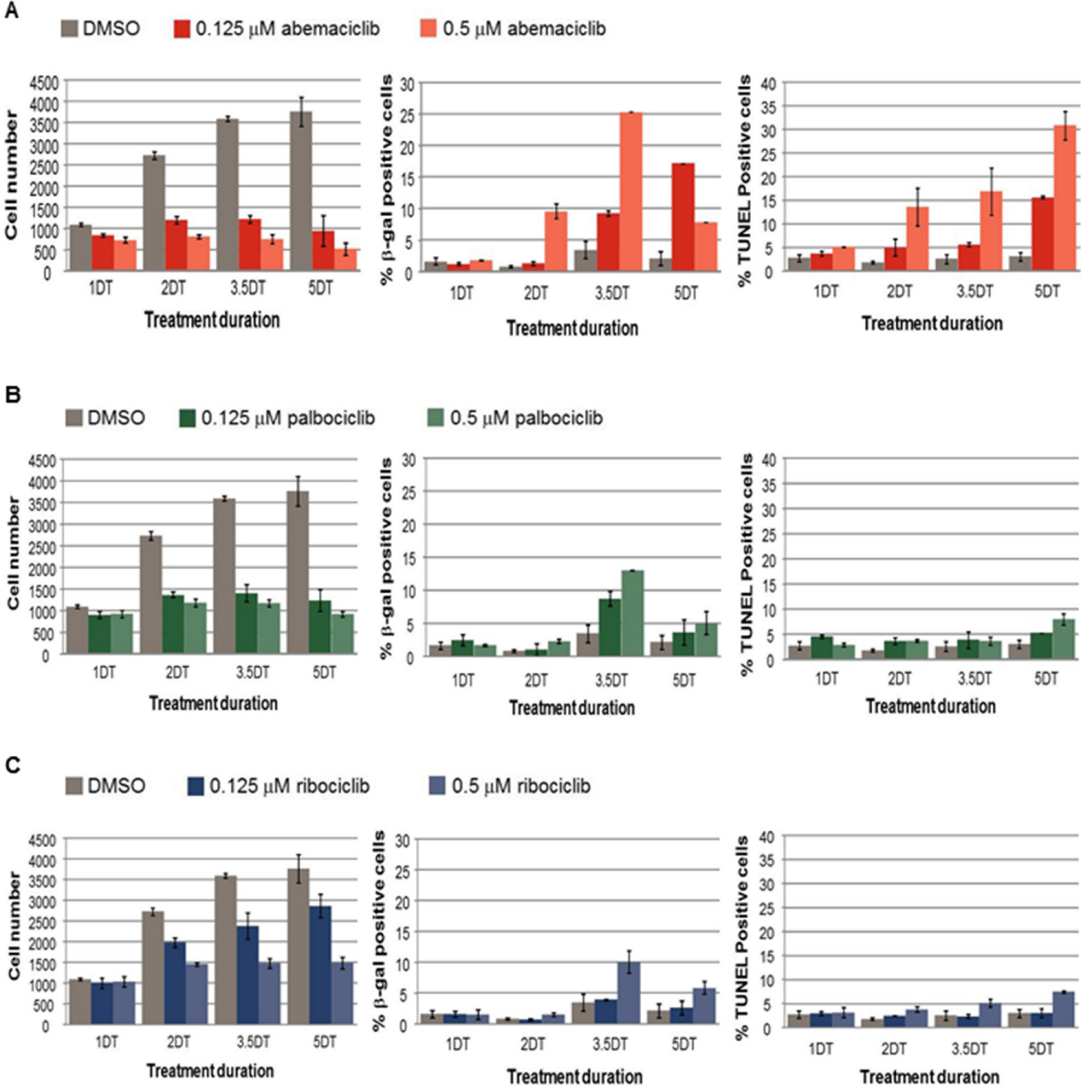
Cell number, senescence and DNA fragmentation in MCF7 cells treated with abemaciclib, palbociclib, or ribociclib **(A–C)** MCF7 cells were treated with DMSO, 0.125 µM or 0.5 µM of the CDK4 and CDK6 inhibitors abemaciclib (A), palbociclib (B), or ribociclib (C). The viable cell number was determined by PI staining, the % of β-gal positive cells was determined using an antibody to β-gal, and the % of Tunel positive cells was determined by the incorporation of labelled dUTP. The data are plotted as the mean (+/- SD) of 2 experiments for CDK4 and CDK6 inhibitor treatment, and the mean of 6 experiments (+/- SD) for untreated samples. The duration of treatment is plotted along the x-axis in doubling times (DT) for MCF7 cells.

Next, the fragmentation of DNA ends was assessed using TUNEL staining, concurrently with senescence and total viable cell number A decrease in cell proliferation concurrent with an increase in percentages of β-gal positive and TUNEL positive cells was observed in MCF7 cells treated with abemaciclib in a time and concentration-dependent manner, with the exception that β-gal levels decreased at the highest concentration of abemaciclib at 5 DT in MCF7 cells (Figure 5A). Under the conditions tested, a similar decrease in viable cell number was observed with treatment of palbociclib compared with abemaciclib; however, the percentage of cells with β-gal staining was lower for palbociclib (as well as ribociclib) than for abemaciclib (Figure 5). An increase in TUNEL staining was observed by 2DT for cells treated with abemaciclib, while only minor increases in TUNEL staining were observed and only after longer treatment times in cells treated with palbociclib and ribociclib (Figure 5). Taken together, the data suggest that abemaciclib leads to the cell cycle arrest of ER+ breast cancer cells, and which becomes irreversible to the induction of senescence and apoptosis.

### Abemaciclib promoted metabolic alterations in metastatic HR+ breast cancer cell lines

**Figure 6:**
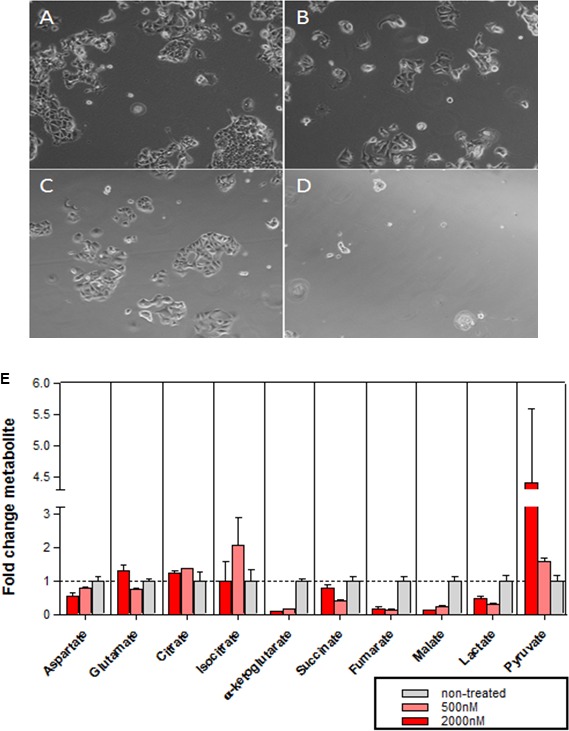
Effects of abemaciclib on cellular metabolism **(A–D)** Glutamine-dependence of MCF7 cell growth. Microscopy images of MCF7 cells grown for 2.5DT in complete DMEM with DMSO 0.2% (A), complete DMEM with abemaciclib 0.5µM (B), glutamine starvation medium with DMSO 0.2% (C), or glutamine starvation medium + abemaciclib 0.5µM (D). (**E**) Metabolic profile in MCF7 cells after 3DT of treatment with abemaciclib at 500 nM and 2000 nM. The mean of duplicate assays is shown (+/- SD) for a representative experiment; the experiment was repeated on 4 separate occasions.

The growth of MCF7 cells was decreased under glutamine starvation, an effect that was enhanced upon concurrent treatment with abemaciclib (Figure 6A–6D, Supplementary Figure 4). Metabolic profiling was carried out in MCF7 cells treated with abemaciclib at 0.5 and 2 µM, over a time course, to profile glutamate and several additional metabolites including aspartate, citrate, isocitrate, α-ketoglutarate, succinate, fumarate, malate, lactate and pyruvate (Figure 6E and Supplementary Tables 1, 5). The level of metabolites began decreasing at 1 DT and 2 DT (data not shown), and became more pronounced at 3 DT. A drop in the concentration of the metabolites α-ketoglutarate, fumarate and malate was observed after treatment with 0.5 µM abemaciclib in relation to vehicle-treated cells, and a minor decrease in succinate concentration was also observed, which may be related to alterations in mitochondrial function (Figure 6E and Supplementary Table 5). Α concentration-dependent decrease in α-ketoglutarate was observed at abemaciclib concentrations ranging from 20 µM to 32 nM (Supplementary Table 5).

### Antitumor activity of abemaciclib *in vivo*

**Figure 7:**
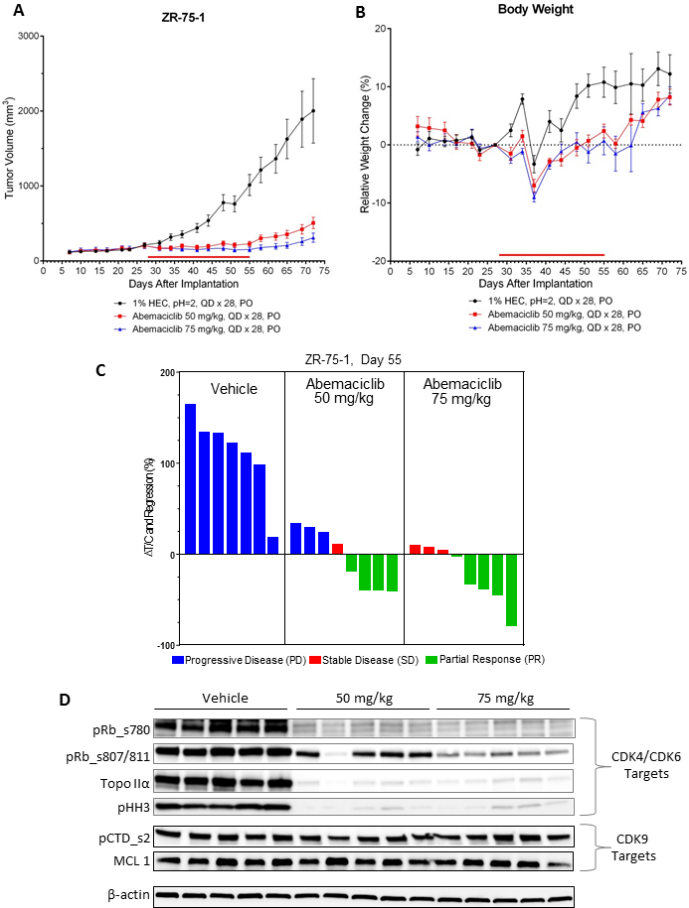
Abemaciclib monotherapy induces regression of ER+ breast cancer xenografts **(A)** NOD SCID mice (*n =* 8/group) implanted with ZR-75-1 xenograft tumors were treated by oral gavage (PO) once-daily for 28 days (QDx28) with 50 or 75 mg/kg of abemaciclib mesylate beginning on day 28 after tumor implantation. Control animals were treated with vehicle (1% HEC/ 25 mM phosphate buffer, pH = 2) using the same schedule of dosing. The red bar just above the X-axis illustrates the duration of the treatment period. Tumor volume measurements were taken routinely during the course of the study and are plotted as mean +/- SEM versus days following tumor cell implantation. (**B**) Body weights were recorded routinely during the course of the study and are plotted as mean % change (+/– SEM) relative to the mean body weight recorded on the day just prior to the first day of dosing (baseline day = 27). (**C**) The waterfall plots indicate the relative tumor volumes for individual animals which were determined at the end of the dosing period (study day 55). The text at the top of each panel of bars provides a description of each of the treatment groups. Growth inhibition is observed in those instances where the calculated changes in %DT/C are less than 100% whereby greater inhibition results in smaller %DT/C values. Change in %DT/C values that are less than zero indicate regressions whereby the tumor volumes on day 55 are smaller than the baseline tumor volume measured on the day prior to the initiation of dosing (baseline day = day 27). The bars for the individual animals are colored on the basis of overall response such that the blue bars indicate progressive disease (PD), the red bars indicate stable disease (SD), and the green bars indicate a partial response (PR). The tumor volumes measured on day 27 served as the baseline volumes for the calculation of %DT/C. Note that one animal in the vehicle control group was sacrificed prior to day 55 due to tumor growth that exceeded the ethical size limits. (**D**) Mice (n = 5/group) implanted with ZR-75-1 xenograft tumors were treated once-daily for 5 days (QD x 5) with vehicle or 50 or 75 mg/kg of abemaciclib beginning on Day 27 after tumor implantation. The xenograft tumors were collected 24 hours later and analyzed by immunoblotting for inhibition of CDK4/CDK6 by assessing the inhibition of phosphorylated Rb (pRb) at serine sites 780 (ser780) or 807/811 (ser807/811), Topo IIα, and phosphorylated histone H3 (pHH3) at serine 10 (ser10). Potential CDK9 inhibition was assessed by measuring the expression of MCL1 or phosphorylation of serine 2 on the C-terminal domain repeat on RNA polymerase II (pCTD). Images of Western blots were cropped to denote the relevant band(s) for clarity; β-actin was used as a loading control.

The *in vivo* anti-tumor activity of abemaciclib was assessed in mice implanted with ZR-75-1 xenograft, a model for human luminal ER+ breast cancer. Dose-dependent anti-tumor activity was observed following treatment with abemaciclib as single agent, with a measured reduction in tumor volume (regression) at the highest dose (75 mg/kg) (Figure 7A–7C). Notably, the analysis of the tumors isolated from mice treated for 5 days showed that the inhibition of tumor growth correlated with inhibition of phospho-(Ser780/807/811)-Rb as well as the inhibition of cell cycle progression as indicated by the inhibition of the expression of Topo IIα and phospho-(Ser10)-H3 observed on Western blots (Figure 7D). The lack of inhibition of either phosphorylation of serine 2 on the C-terminal domain (pCTD) repeat on RNA polymerase II or MCL1 expression, indicated that CDK9 was not inhibited at any dose of abemaciclib (Figure 7D), thereby providing additional evidence for antitumor activity through inhibition of CDK4 and CDK6.

This specificity of abemaciclib for CDK4 and CDK6 is further supported by mRNA expression studies which showed that treatment with abemaciclib resulted in a significant inhibition of expression of various genes involved in cell cycle regulation (Supplementary Figure 3). In particular, treatment with either 50 or 75 mg/kg abemaciclib resulted in the suppression of the expression of several genes encoding for proteins required for progression through the S or G2/M phases of the cell cycle including Cyclin E, MCM7, CDKN2C (p18), PTEN, Aurora A (AURA), cyclin B1 (CCNB1), FOXM1, ribonucleotide reductase subunit M2 (RRM2), Ki67 (MKI67) and topoisomerase II-alpha (Topo IIα). Notably the transcription of these genes, with the exception of PTEN and AURA, are dependent upon E2F1, a transcription factor that is negatively regulated by Rb. Since Rb is the principal kinase target for CDK4 and CDK6, these results provide further evidence that cell cycle inhibition and subsequent inhibition of growth by abemaciclib resulted directly from the inhibition of these two kinases. Together, the data show that abemaciclib is a CDK4 and CDK6 inhibitor that demonstrates both *in vitro* and *in vivo* antitumor activity in breast cancer models.

## DISCUSSION

Several CDK4 and CDK6 inhibitors are currently being studied in clinical trials, including abemaciclib, and FDA-approved palbociclib and ribociclib [13–15, 24]. Here, we explored the detailed mechanisms of action of abemaciclib as a single agent in ER+ breast cancer cell lines and *in vivo*. In cell-free biochemical assays, abemaciclib potently inhibited the kinase activity of both CDK4 and CDK6, with a 14-fold higher potency for CDK4/cyclinD1 complexes than CDK6/cyclinD3. The difference in potencies between these CDK4 and CDK6 complexes with specific D-type cyclins may contribute, to the differential anti- proliferative activity of abemaciclib observed in cell lines derived from various tissue types where distinct CDKs or D-type cyclins may play a distinct role [25]. CDK4 and Cyclin D1 may be especially relevant for breast cancer, since evidence suggests that both are important oncogenic drivers for this disease [4, 9, 10, 12, 26]. As previously described, abemaciclib is specific for CDK4 and CDK6 [16], a result which is confirmed in this current study in breast cancer cell lines.

Treatment with abemaciclib inhibited cell proliferation and led to G1 cell cycle arrest in a concentration-dependent manner, concurrent with inhibition of Rb phosphorylation and of expression of the biomarkers Topo IIα and pHH3; after longer term treatment (more than 2 DT) These changes were maintained after compound removal, in cells treated with abemaciclib for at least 2DT. Consistent with these observations, longer treatment of breast cancer cells with abemaciclib was associated with a loss of replicative capacity, particularly for HR+ compared with HR- breast cancer cells. The higher anti- proliferative activity of abemaciclib in HR+ cells is consistent with known alterations in many of these cell lines, wherein CCND1 amplification indicates that HR+ breast cancers may be especially reliant on this pathway for tumorigenesis [27].

Treatment with abemaciclib induced a concentration- and time-dependent senescence response, as shown by multiple markers, including the formation of heterochromatin foci and β-gal expression. Additionally, apoptosis was observed in Rb-proficient breast cancer cell lines following prolonged treatment with abemaciclib, as indicated by annexin V and TUNEL. Promotion of apoptosis after treatment with CDK4 and CDK6 inhibitor has been described in leukemic cells [6]. Continuous, long-term treatment with abemaciclib also altered primary cell metabolism as indicated by the observed TCA metabolite profiles as well as increased cell death upon glutamine deprivation. Thus, abemaciclib treatment induced potent, stable inhibition of breast cancer proliferation, most likely through the induction of long-term sustained changes in cells including senescence and apoptosis.

Abemaciclib induced tumor regression *in vivo* in xenografts of HR+ breast cancer, with corresponding changes of molecular markers of cell cycle inhibition, including phosphorylation of Rb and TopoIIα expression in tumors from mice administered abemaciclib. Transcriptional profiling studies from tumors isolated from mouse xenografts of breast cancer after abemaciclib treatment, show that growth inhibition by abemaciclib correlates with inhibition of expression of genes which are dependent on the E2F family of transcription factors for expression. The expression levels of FOXM1, RRM2, TOPO2A, MKI67, MCM7, CDKN2C, CCNE1 (Cyclin E1), and CCNB1 (cyclin B1) mRNA were all significantly reduced following treatment with abemaciclib (Supplementary Figure 3). These results are again consistent with the well-defined roles of CDK4 and CDK6 as kinases which reverse the Rb-mediated suppression of E2F-dependent transcription. Known targets of CDK9, including the CTD of RNA Polymerase II and expression of MCL1, were not affected by abemaciclib either in cell lines or in xenograft tumors, further indicating that CDK4 and CDK6 are the principal targets of abemaciclib.

The current studies indicate that abemaciclib induces concentration-dependent changes in cellular metabolism, senescence and apoptosis which are time-dependent indicating that the ability to maintain sustained inhibition of CDK4 or CDK6 in addition to the potent inhibition of these targets may be essential for achieving optimal efficacy. Interestingly, the loss of replicative capacity in HR+ breast cancer cells occurred earlier upon treatment with abemaciclib when compared to treatment with the same concentration of palbociclib (Supplementary Figure 1). Moreover, both senescence and apoptosis occurred earlier and at lower concentrations of abemaciclib, compared to treatment with either palbociclib or ribociclib (Figure 5). The results of the studies presented here suggest a relationship between the potencies of CDK4 and CDK6 inhibitors and the onset of senescence. Promotion of senescence after treatment with CDK4 and CDK6 inhibitors or CDK4 ablation has been described in other cancer cell types [6, 7, 8]

Altogether, the data demonstrate that abemaciclib was effective at blocking breast cancer cell progression and viability through multiple mechanisms which include cell cycle arrest, the induction of senescence as well as upon prolonged exposure, apoptosis and altered energetic metabolism. Moreover, these studies also provide some preliminary mechanistic insights regarding the single-agent activity that has been observed in clinical trials with abemaciclib [17, 18], and may distinguish this particular CDK4 and CDK6 inhibitor from other CDK4 and CDK6 inhibitors currently in clinical use or undergoing clinical evaluation.

## MATERIALS AND METHODS

### Ethics statement

All animal studies were performed in accordance with American Association for Laboratory Animal Care institutional guidelines, and all protocols were approved by the Eli Lilly and Company Animal Care and Use Committee.

### Synthesis and naming of compounds

All compounds were synthesized at Lilly Research Laboratories (LRL). Unless otherwise indicated, all preclinical data described herein for abemaciclib and palbociclib were obtained using the methanesulfonate salt of each compound; ribociclib was synthesized as a succinate salt. Full chemical names have been previously published [16]. The PI3K/mTOR inhibitor BEZ235, dactolisib [2-Methyl-2-[4-(3-Methyl-2-oxo-8-quinolin-3-YL-2,3-dyhydro-1H-imidazo[4,5-C]quinolin-1-YL)phenyl]propanenitrile] was synthesized and characterized for purity and identity at LRL.

### Antibodies

Mouse anti-Rb antibodies from BD Pharmigen (cat# 558385), or Cell Signaling Technology (cat# 8516s, or 9308s) were used for detection of Rb phosphorylated at S780, or S807/811, respectively. Other antibodies used were: topoisomerase (topo) II α (Cell Signaling Technology, cat# 12286s), histone H3 (tri methyl K9) (Abcam cat# ab8898), phospho-Histone H3 (Ser10) (Millipore cat# 06-570), phospho-CTD (Abcam cat# 5095), Mcl-1 (Cell Signaling Technology cat# 5453), FOXM1 (Abcam cat# ab175798), Ki67 (Abcam cat# ab16667), β-galactosidase (Abcam cat# ab9361), goat anti-mouse IgG-Alexa Fluor^®^ 488 conjugated antibody (Life Technologies cat# A11017), goat anti-rabbit IgG-Alexa Fluor^®^ 488 conjugated antibody (Life Technologies cat# A11008), and goat anti-chicken IgY H&L-Alexa Fluor^®^ 647 conjugated antibody (Abcam cat# ab150171). For TUNEL assays, Roche cat#11767291910, 11767305001 and 11966006001 were used, and an annexin V-FITC kit was used for flow cytometry (Miltenyi Biotec cat# 130-092-052).

### Cell lines and culture conditions

EFM-19 cells were purchased from DSMZ (#ACC 231) and grown in RPMI-1640 medium (Gibco #52400), supplemented with 10% FBS (Gibco #16000) and 1% Penicillin/Streptomycin (Gibco #15140-122). MCF7 (#HTB-22), T-47D (#HTB-133) and MDA-MB-361 (#HTB-27) cells were purchased and authenticated by ATCC. MCF7 were grown in DMEM (Sigma #D5796), supplemented with 10% FBS, 1% Penicillin/Streptomycin and 0.01 mg/mL human insulin (Sigma #I9278). MDA-MB-361 were grown in RPMI-1640, supplemented with 20% FBS and 1% Penicillin/Streptomycin; additional information can be found in the Supplemental Methods. Except indicated otherwise, all cell lines were grown as indicated by the ATCC. For most experiments, cells were plated (prior to becoming 70% confluent) in 96-well flat-bottom plates at a density of 1000 cells per well in 100 µL (exceptions are indicated below). Cells were incubated overnight in a cell culture incubator (5% CO_2_, 95% Relative Humidity (RH) and 37°C) and allowed to attach to the plate (approximately 14–24 hours after seeding) before treatment. The doubling times (DT) of cell lines were determined by growing cells in 6-well plates and counting cells every 24h after seeding using Vi-cell. Cell number was obtained and fitted to the following exponential growth equation regression: DT=T ln2/ln(Xe/Xb); where T is the incubation time, Xb is the cell number at the beginning of the incubation time and Xe is the cell number at the end of the incubation time [19]. Doubling times were calculated to be approximately 48 hours (2 days) for MCF7 cells, 72 hours (3 days) for EFM-19 cells and 120 hours (∼5 days) for MDA-MB-361 cells.

### Determination of IC_50_/EC_50_ values

Relative IC_50_/EC_50_ values were determined by curve fitting to a four parameter logistic equation: Y = bottom + ((top-bottom)/ (1+exp(HillSlope*(X – LnIC_50_)), where X is the natural log of dose/concentration, Y is response, decreasing as X increases, top and bottom are plateaus in the same units as Y, lnIC_50_ is the natural log of potency, in the same log units as X, and HillSlope measures the steepness of the curve [20]. Data were plotted using GraphPad Prism.

### Enzyme kinetics

Kinetic parameters for CDK4/cyclinD1 and CDK6/cyclinD1 complexes as well as IC_50_ values for CDK9/cyclinT1 were obtained as previously described [16]. Additional details for CDK6/cyclinD3 activity are provided in Supplemental Methods.

### High content imaging cell based assays for monitoring phosphorylation of Rb (Ser780), G1 arrest and cell proliferation

MDA-MB-361 or EFM-19 cells were incubated for 16 hours with 10 different concentrations of abemaciclib (starting with 20 µM and using a 3-fold dilution series down to 0.001 µM). As controls, cells were treated with 10 µM palbociclib or vehicle ( 0.2 % v/v DMSO). Additional details for cell-based assays are provided in the Supplemental Methods.

### Cell immunolabeling with 5-bromodeoxyuridine (BrdU)

MCF7 cells were plated in a 96-well format and treated for 3 DT with 2 µM (or 3-fold dilutions) of abemaciclib. After treatment, an ELISA for BrdU detection was performed (Roche, Cat. No. 11 669 915 001) following the vendor’s manual. Envision^®^ (PerKinElmer) was used to measure the light emission of the samples and raw data were processed with Microsoft Excel and relative EC_50_ values were determined as described above. For normalization, the mean value obtained from cells treated with 2 µM µM doxorubicin (Sigma #D1515) was set as the minimum, and that obtained from treatment with the vehicle (0.2% DMSO) was set as the maximum.

### FOXM1 and Ki-67 protein expression

EFM-19 cells were treated with 2 µM (or a series of 5 additional 3-fold dilutions) of abemaciclib. After 72 hours, cells were stained with an anti-FOXM1 antibody and a secondary goat anti-rabbit IgG, Alexa Fluor^®^ 488 conjugate; nuclei were stained with propidium iodide (PI). Alternatively, for assessment of Ki-67 levels, cells were treated for 2 DT and then stained with Ki-67 and a secondary goat anti-rabbit IgG, Alexa Fluor^®^ 488 conjugate. Plates were read in an Acumen Explorer Ex3 (TTP Labtech) using Cellista software and the percentages of FOXM1 positive and Ki-67 positive cells were calculated over the background signal. Data were normalized in Excel using the mean value obtained from treatment with 2 µM BEZ235 as the minimum and the mean value obtained from treatment with 0.2% DMSO as the maximum.

### Side scatter and detection of annexin V by flow cytometry

Changes in complexity and morphology of cells treated with 0.2% DMSO or 0.5 µM of abemaciclib 3 DT for MCF7, EFM-19, or MDA-MB-361 were monitored by side scatter (SSC-A) measurements using a MACSQUANT^®^ Analyzer 10 (Miltenyi Biotec). Annexin V/PI staining was performed following the vendor’s specifications in MCF7 and MDA-MB-361 cells. Cells were treated with 0.2% DMSO or 0.5 µM and 2 µM of abemaciclib overnight for 3 DT, and assessed by flow cytometry (MACSQuant^®^ Analyzer 10, Miltenyi Biotec). Raw data were analyzed with FlowJo X software. Events were gated for debris exclusion and singlets selection. A minimum of 10,000 cells were analyzed per sample.

### SA-β-gal immunofluorescence

MCF7 cells seeded in 24 well plates were treated with 0.5 µM, or 2 µM of abemaciclib or vehicle (0.2% v/v DMSO) and incubated for 4, 7 or 10 days for single detection of β-gal. For multiplexing experiments, cells seeded in 96 wells plates treated with 0.5 µM or 0.125 µM of abemaciclib or vehicle and treated for 1, 2, 3.5, or 5 DT. After treatment, cells were fixed, washed with PBS, permeabilized and β-gal was detected using anti-β-galactosidase antibody; for multiplexed assays, TUNEL staining was carried out using Roche TUNEL reagents as indicated above. Nuclei were also stained with PI for total cell counting. Plates were read in an Acumen Explorer Ex3 (TTP Labtech) and raw data were analyzed with Cellista software. Outputs were expressed as percentages of each identified subpopulation 1) positive β-gal, and 2) total cell number. The percentages of β-gal positive cells, TUNEL positive cells, and cell numbers were plotted in Microsoft Excel.

### TCA metabolite profiling by LC/MS

Cells were incubated with a 5-fold dilution series of abemaciclib ranging from 20 µM to 0.032 µM final concentration in 0.4% v/v DMSO; vehicle-treated cells (0.4% v/v DMSO in growth media) were used as a control. MCF7 cells were plated at 20,000 cells/well in 24-well plate; approximately 14–24 hours after seeding, cells were treated with DMSO (vehicle) or the indicated compounds diluted in DMSO. Cells were incubated for three different incubation times: 48 hours (1 DT), 96 hours (2 DT), and 6 days (3 DT). After treatment, cells were harvested and the collected pellet was stored at -80°C. For additional details see Supplemental Methods and Supplementary Table 1.

### Mouse xenograft tumor implantation and treatment

Female NOD SCID mice (Harlan) 18–20 grams in size were used for these studies. For subcutaneous tumors, growth was initiated by subcutaneous injection of 5 × 10^6^ human ZR-75-1 cells in a 1:1 mixture of HBSS and Matrigel™ (BD Bioscience, Franklin Lakes, NJ) in the rear flank of each subject animal. Since growth of this model is dependent upon a source of estradiol, the mice were implanted with 17-B estradiol 90-day release pellets (0.72 mg, Innovation Research of America) 1 day prior to the implantation of the tumor cells. When mean tumor volumes reached approximately 200 mm^3^ in size, the animals were randomized by tumor size and body weight and placed into their respective treatment groups. Abemaciclib (lot# E29-H71350-002) was formulated in 1% hydroxyethyl cellulose (HEC) in 25 mM phosphate buffer (PB) pH = 2. For the efficacy studies, abemaciclib was administered by oral gavage once-daily for 28 days (PO, QD × 28) at doses of 50 or 75 mg/kg using 0.2 mL/dose. The control group was given 1% HEC vehicle according to the same schedule for abemaciclib. A parallel set of tumor-bearing mice used for the *in vivo* target inhibition (IVTI) studies were treated daily with the same doses of abemaciclib for 5 days. Additional details are provided in Supplemental Methods.

## SUPPLEMENTARY MATERIALS FIGURES AND TABLES


