# Supplementary material for: Preclinical characterization of abemaciclib in hormone receptor positive breast cancer
Source: Oncotarget. 2017 May 10;8(41):69493–507. doi: 10.18632/oncotarget.17778 (PMC5642494; doi:10.18632/oncotarget.17778)
Supplement: Supplementary file 1 [file oncotarget-08-69493-s001.pdf]

Preclinical characterization of abemaciclib in hormone receptor positive breast cancer

Supplementary Materials

SUPPLEMENTALRY METHODS

Enzyme kinetics of CDK6/cyclin D3 complexes

CDK6/cyclin D3 activity was measured by the phosphorylation of CTRF using [g-33P] ATP at 20°C in a 96-well plate filtration assay format. The enzyme reaction was conducted in final a volume of 50 µL in a kinase buffer containing 68 mM Hepes at pH 7.5, 6.67 mM $MgCl_2$, 6.67 mM dithiothreitol (DTT), 0.003% (w/v) BSA and 0.013% TRITON™ X-100. 10µL of CDK6/cyclin D3 enzyme complex (Millipore 14-519, #D13BP009NA) (1 nM final concentration in the assay) was added onto a mix of substrate and test compound. Concentration of compounds ranged from 1 µM to 0.1 nM and ATP varied from 1 mM to 7.8 µM (1.12 mCi [^{33}P] –ATP per µmol ATP) (PerkinElmer #NEG602K250UC) and a constant concentration of 0.8 µM C-Terminal Retinoblastoma Fragment (CTRF) was used (Upstate #12-439). The reaction was carried out in a 96-well plate with 4% (v/v) DMSO buffer as a control to obtain an ATP saturation curve and K_M^{ATP}. 125 mM ethylene diamine tetraacetic acid (EDTA) was used to determine the level of background ^{33}P in the absence of enzyme activity. Reagents were mixed and incubated for 30 min at 20 ºC. The reaction was terminated by the addition of 80 µL 10% (v/v) H_3PO_4 and precipitation of material onto glass fiber filter plates (Millipore, MAFC N0B 50). The wells were washed 4 times with 0.5% H_3PO_4 and the radioactivity incorporated was measured with a microplate scintillation counter (Microbeta Trilux, Wallac).

Kinetic data analysis

Data obtained were fitted to the Michaelis-Menten equation for a competitive inhibitor (Copeland 2000) using GraphPad Prism.

$$V = \frac{v_{MAX}[ATP]}{K_M obs + [ATP]} \text{; where } K_M obs = K_M\left(1 + {[I]}/{K_i}\right)$$

Data reported are the results of averaged values obtained in independent duplicate experiments.

High content imaging cell-based assays for monitoring phosphorylation of Rb (ser780), G1 arrest and cell proliferation

Cells were fixed with para-formaldehyde and Rb phosphorylation at Serine 780 was detected using a primary mouse anti-Rb (PS780) antibody followed by a secondary goat anti-mouse IgG-Alexa Fluor 488 conjugated antibody; cells were concurrently stained with propidium iodide (PI). Plates were read in an Acumen Explorer eX3 (TTP Labtech). The pRb (Ser780) positive subpopulation was identified using the channel corresponding to 500-530 nm and the percentage of cells with pRb signal relative to the total PI stained subpopulation was calculated by the instrument's software (Cellista, TTP Labtech). Raw data were then normalized in Microsoft Excel to obtain the percentage of pRbSer780-positive cells using the following equation: % of cells positive for pRb780 = (signal – $Mean_{min}$)/($Mean_{max}$ – $Mean_{min}$) × 100, where the mean value obtained from cells treated with palbociclib was set as the minimum and the mean value obtained from vehicle-treated cells was set as the maximum subpopulation.

G1 cell cycle arrest and cell proliferation were evaluated by measuring PI signal using high content imaging. MDA-MB-361 or EFM-19 cells were incubated with a final concentration of 20µM (or a 3-fold dilution series) of abemaciclib with the treatment covering at least 2 DT of each cell line. As controls, cells were treated with 1µM nocodazole or DMSO. Cells were fixed with Prefer (Anatech Ltd# 402) and nuclei were stained with PI. Plates were read in an Acumen Explorer Ex3 (TTP Labtech) and the percentage of cells in the G1 phase and the total number of cells per well were determined by the Cellista software. Assay outputs were calculated by the Cellista software as a percentage of each identified subpopulations, % pRb, % 2n, % 4n, and total cell number. Cell proliferation raw data were further normalized using the formula described for analysis of pRb signal, except that the minimum was set as the mean signal obtained from cells treated with nocodazole; data were plotted using GraphPad Prism.

Additional cell line information

The following cell lines were obtained from ATCC and were tested by the CellCheck 9 assay (IDEXX

BioResearch) where indicated; the dates of acquisition and testing are listed in parentheses: ZR-75-1; T-47D; MDA-MB-468; HCC70; BT-549; BT-20; MDA-MB-231; MDA-MB-175-VII, HCC-1937; MDA-MB-415.

SA-β-galactosidase immunocytochemical labeling

MCF7 cells were seeded in a 24-well plate, incubated overnight and then treated for 4 days with DMSO 0.2% or with 0.5 μM of abemaciclib. SA-β-gal expression was determined using the Senescence Cells Histochemical Staining Kit (Sigma, CS0030) following the vendor's manual. Images were taken at 100x by bright field microscopy (Leica DMiL).

Proliferation studies after washouts (EdU Click-iT®)

Cells were plated onto poly-D-lysine coated 96-well plates at a density of 2,000–6,000 viable cells per well, and treated with abemaciblib or palbociclib at 1μM following the cell treatment and washout schedule shown on Supplementary Figure 1A. An EdU (5-ethynyl-2'-deoxyuridine) pulse was added one hour prior to the end of study at a final concentration of 10 μM as described in the kit protocol (Click-iT High Content Imaging EdU Incorporation kit (Life Technologies cat# C10351)). The cells were then fixed in the presence of 3.7% formaldehyde for 20 minutes at 37°C, and permeabilized with 0.1% Triton-X 100 in PBS for 10 minutes at 25°C, then washed twice with PBS. They were blocked using 1% bovine serum albumin (BSA) (Invitrogen #15260-037) for 1 hour at 25°C. EdU detection was performed using the Click-iT High Content Imaging EdU Incorporation kit (Life Technologies cat# C10351) and Hoechst33342 stainning to detect nuclear material (Molecular Probes #21492). Cell images were captured using a Cellomics Arrayscan VTI and analyzed with the Target Activation bio-application V.3 reading in 4 channels at a magnification of 10X.

Objects were identified using an algorithm to detect nuclear staining with Hoechst 33342 dye. The relative levels and sub-cellular localization of EdU were determined through the respective intensities and locations of Alexa-488 fluorescence.

Assessment of hypermethylation of histone H3 at lysine 9 (H3K9me3)

MCF7 and EFM-19 cells were plated in 96-well plates and treated at 20 μM (or a 3-fold dilution series) of abemaciclib for 2 DT for each cell line in duplicate; a Tecan Genesis RSP100 liquid-handling system was used to administer the dilution series to cells. After treatment, IHC staining was conducted with an H3K9me3 antibody and a secondary goat anti-rabbit IgG, Alexa Fluor® 488 conjugate; nuclei were stained with PI. Plates were read in an Acumen Explorer Ex3 (TTP Labtech), raw data were analyzed with Cellista software and then further normalized in Excel against the values obtained for cells treated with 2 μM BEZ235, or vehicle (0.2% v/v DMSO); data were graphed using GraphPad Prism®. Images were taken by fluorescent microscopy using a Leica DMiL.

LC-MS/MS sample treatment

Cell samples were lysed after treatment with 100 μL lysis buffer and transferred to a 96-well plate. 30 μL of each sample were transferred into deep well plates using TECAN-EVO Workstation. Samples were treated for derivatization of the metabolites of interest and internal standards. For this, 50 μL of derivatization reagent and 80 μL of internal standard (IS, SupplementaryTable 1), were added with multidrop, plates were vortexed for 5 mins and let sit for one hour at 4ºC. The IS used were the D or ^{13}C-labeled commercial analogues of nine of the ten metabolites, prepared at 25 μM in water (Supplementary Table 1). The derivatization reagents (from Sigma), 3-nitrophenylhydrazine (0.5 M) and the coupling buffer [N-(3-Dimethylaminopropyl)-N′-ethylcarbodiimide hydrochloride (1M) were freshly prepared in 75:24:1 methanol/water/pyridine.

After derivatization, a liquid-liquid extraction step with ethyl acetate was carried out in two steps; the upper phase was transferred to a new deep-well plate with TECAN-EVO, and the joint extract was evaporated to dryness with nitrogen, and reconstituted with 50% H2O/MetOH. A set of calibrants at 12 levels of the analytes was derivatized in parallel following the same protocol. Samples were analyzed by LC─MS/MS under the conditions described below.

LC/MS/MS Sample Analysis

Analysis of the derivatized-extracted samples was carried out with an Agilent 6460 Triple Quadrupole Mass Spectrometer (qQq) with JetStream Electrospray Ionization (ESI) Source, coupled to a LC1290 Infinity High-Performance Liquid Chromatograph. The software for data collection and treatment was Agilent Masshunter Workstation. Mobile phases for chromatography were: mobile phase A: formic acid (5 mM) and ammonium formate (10 mM) in water; mobile phase B: acetonitrile. 50% Acetonitrile/water were used to clean the injection needle after each injection. A reverse-phase chromatographic column (XBridge BEH C18 3.5 μm, 2.1 × 50 mm) from Phenomenex (Torrance, CA, USA) was used as stationary phase. Chromatographic separation was performed as follows: a gradient from 95% to 40% of mobile phase A in 6 min; then to 5% in 0.1 min, maintained 0.7 mins. The total analysis time was 6.8 min, followed by 1 min of re-equilibration with initial mobile phase.

Analyses were carried out in multiple reactions monitoring (MRM) negative ionization mode with nitrogen

as drying and nebulizing gas. The operating conditions of the ESI–QqQ, were: flow rate and temperature of drying gas 9 mL/min and 350ºC, nebulizer pressure 45 psi, capillary voltage 4000 V, nozzle voltage 500V delta EMV (potential of the electron multiplier) 400 V, and sheath gas temperature and flow rate 300ºC and 12 mL/min, respectively. Analysis conditions for each analyte and its internal standard are in SupplementaryTable 1.

Agilent MassHunter quantitative software was used for quantitative analysis; calibration curves for each analyte were obtained from the area of the analyte to the area of internal standard ratio. Calibration curves for each analyte were obtained from the relative area of analyte to the internal standard vs concentration, by 1/x weighted least squares linear regression. Concentrations are calculated by extrapolation of the analyte area to IS area into the corresponding calibration curve. Raw concentrations of each metabolite in treated cells were normalized to concentration of metabolites in controls, which were set to 1. Data were processed with Graphpad Prism and Microsoft Excel. For the experiments where metabolite concentration is normalized to total protein content, this was measured with Bradford assay kit (Bio-Rad), measuring absorbance at 595nm in Spectramax, as concentration of protein referred to the standard of BSA in lysis buffer (μg/mL).

All experiments were carried out in duplicate or triplicate and were repeated on at least two separate occasions; independent experiments yielded comparable results and a representative example for each experiment is shown.

Additional Methods for Mouse Xenograft Studies

Tumor Data Capture

Tumor size and body weight were captured using Web Director. Tumor volume (V) was estimated by using the formula: $V = 0.536L \times W^2$ where L = larger of measured diameter and W = smaller of perpendicular diameter. The tumor volume data were transformed to a log scale to equalize variance across time and treatment groups. The log volume data were analyzed with a two-way repeated measures analysis of variance by time and treatment using the MIXED procedures in SAS software (Version 9.3). The correlation model for the repeated measures was Spatial Power. Treated groups were compared to the control group at each time point. The MIXED procedure was also used separately for each treatment group to calculate adjusted means and standard errors at each time point. Both analyses accounted for the autocorrelation within each animal and the loss of data that occurred when animals with large tumors were removed from the study early. The adjusted means and standard errors are plotted for each treatment group versus time.

Relative changes in tumor volume (%DT/C) were calculated using the tumor volume measurements taken at the end of the dosing period with abemaciclib (study day 55), whereas the baseline tumor volume was the volume recorded on the day just prior to first day of dosing (baseline day = study day 27). %DT/C values were calculated using the formula $\%DT/C = 100 \times DT/\Delta C$, whereby T = mean tumor volume of the compound treated group, DT = mean tumor volume of the compound treated group minus the mean tumor volume on the baseline day, C = mean tumor volume of the control (vehicle) group, and ΔC = mean tumor volume of the control group minus the mean tumor volume on the baseline day. If DT was < 0, then a tumor regression value was calculated instead of % DT/C whereby $\%Regression = 100 \times DT/T_{initial}$ such that $T_{initial}$ = the grand mean of the tumor volume for all of the treatment groups.

Growth inhibition is observed in those instances where the calculated values for %DT/C are less than 100% whereby greater inhibition results in smaller % DT/C values. Calculated values of %DT/C greater than 100% indicate instances where the average tumor volume of the treated group is larger than the average tumor volume of the vehicle control group. Any negative values for % DT/C listed in the tables and figures are values for % regression whereby the average tumor volume for the treated group is less than the tumor volume measured on the baseline day (before treatment was initiated). For the waterfall plots, progressive disease (PD) is defined as an increase in % DT/C relative to baseline of ≥ 20%; stable disease (SD) is defined by tumor volumes which show any measureable increase in tumor volume relative to baseline which is < 20% (0% < SD < 20%); a partial response (PR) is defined by the range of tumor volumes which either show no growth relative to baseline (0%) or have reductions in tumor volume of 80% or less (0% ≥ PR ≥ –80%); and a complete response (CR) is defined by reductions in tumor volume greater than 80% (< –80%).

In vivo target inhibition analysis

A set of tumor-bearing mice treated in parallel with those for the efficacy analysis were included for *in vivo* target inhibition (IVTI) studies. For these IVTI studies, 5 mice per treatment group were treated daily for 5 days (QD × 5) using the same doses of abemaciclib for the efficacy study. These mice were then sacrificed 24 hours after the last dose and the ZR-75-1 xenograft tumors were harvested for protein expression by immunoblotting. The harvested tumors were lysed in boiling 1% SDS (plus protease and phosphatase inhibitors) and the total protein concentration in each tumor lysate was quantified using the BCA protein assay kit (Pierce # 23225). The proteins within the lysates were resolved by SDS-PAGE and then transferred to nitrocellulose membranes for immunoblotting with antibodies which recognize phosphorylated Rb (pRb) at serine sites at 780, 807 and 811 (ser780/807/811), Topo IIα, or phosphorylated histone H3 (pHH3) at serine 10 (ser10). Inhibition of pRb at serines 780, 807 or 811 provides a direct measure of CDK4 and/or CDK6 inhibition since both these kinases phosphorylate Rb at these serine sites. Likewise the inhibition of Topo IIα

or pHH3 facilitates quantification of the inhibition of cell cycle progression since lower expression of these 2 biomarkers quantitatively correlates with the extent of progression of cells through either S-phase (Topo IIα) or M-phase (pHH3). The inhibition of CDK9 was assessed by measuring the inhibition of either MCL1 expression or phosphorylation of serine 2 on the C-terminal domain repeat on RNA polymerase II (pCTD). The visualization of the signals corresponding to these biomarkers was achieved through development with the ECL SuperSignal chemo-luminescence kit (Pierce) and image capture using the Fuji LAS400 image capture instrument.

Isolation and purification of RNA from tumors

Frozen tumors were pulverized and then resuspended in complete RLT lysis buffer (10ml BME/mL RLT stock) which was included in kit (RNeasy Mini Kit (Qiagen #79654). The suspension was placed on the Qiashredder columns (Qiagen #79654) and RNA was purified by following the protocol for the RNAeasy Mini Kit (Qiagen # 74104). Following the last wash, the purified RNA was eluted using 50 ml RNase-free water and collected in labeled DNase/RNase-free collection tubes. The quantity of RNA was measured by spectral analysis using the Nanodrop Spectrophotometer (NanoDrop Products, Wilmington, DE, USA) and then placed in -80 degree F freezer for analysis. The cDNA preparation was completed as described in the included kit protocol using Superscript VILO Mastermix (Invitrogen #11755-250) and DEPC-Treated Nuclease Free Water (Ambion, #AM9915G). All samples, once quantitated, were delivered to the Eli Lilly Clinical Diagnostic Laboratory (CDL) for analysis using the Qiagen MODAplex/ICEPlex instrument which combines amplification and capillary electrophoresis for target separation, detection, and analysis.

Supplementary Table 1: Commercial sources and MS/MS analysis conditions for analytes and internal standards

**Analyt**	**Commercial[a]**	**Precursor Ion**	**Fragmentor**	**Product Ion**	**Collision Energy**
α-ketoglutarate	Sigma	550.1	138	233.1	24
Pyruvate	Sigma	357.1	116	137	20
Succinate	Sigma	387.1	130	234.1	12
Isocitrate	Sigma	596.1	136	234	28
Glutamic acid	Sigma	263.1	104	137	16
Fumaric acid	Sigma	385.1	122	234	12
Malic acid	Sigma	403.1	120	208	12
Lactic acid	Sigma	224.1	86	152	8
Citrate	Sigma	596.1	136	222	24
Internal Standard	Commercial[a]	Precursor Ion	Fragmentor	Product Ion	Collision Energy
Succinic acid-2,2,3,3-D4	Sigma	391.1	138	237.1	12
2-Ketoglutaric acid-D6	Sigma	554.1	142	375.1	16
DL-Glutamic-2,4,4-D3 Acid	C/D/N isotopes	266.1	106	138	16
(RS)-Malic-2,3,3-D3 Acid	C/D/N isotopes	406.1	106	209	12
Citric-2,2,4,4-D4 Acid	C/D/N isotopes	600.1			
Fumaric-2,3-D2 Acid	C/D/N isotopes	387.1	132	236	12
Sodium DL-Lactate 2,3,3,3-D4	C/D/N isotopes	228.1	138	152	8
Sodium pyruvate-D3	Cambridge Isotope Laboratories	360.1	134	150	16

[a]Sigma (St. Louis MO, USA), C/D/N Isotopes (Quebec, Canada), Cambridge Isotope Laboratories (Andover, MA, USA).

Supplementary Table 2: Summary of cellular G1 arrest, pRb and cell proliferation inhibition potency in luminal ER^+ cell lines

	Abemaciclib IC50 (µM)		
**Breast Cancer Cell Line**	**pRb (Ser780)**	**2n**	**Cell number**
EFM-19	0.012 ± 0.011	0.032 ± 0.021	0.019 ± 0.004
MCF7	0.013 ± 0.002	0.054 ± 0.016	0.369 ± 0.37
MDA-MB-361	0.067 ± 0.037	0.038 ± 0.002	0.045 ± 0.014

IC_{50} values were obtained from a 10-point dose response experiment and are presented as Arithmetic Mean ± Stdev (n = 2).

Supplementary Table 3: Percentage of β-galactosidase positive MCF7 cells after treatment with abemaciclib

	% β-gal Positive MCF7 Cells[a]		
		Abemaciclib concentration	
**Treatment Time**	**DMSO**	**0.5 µM**	**2 µM**
4 days	1.54 ± 0.41	16.15 ± 1.48	42.45 ± 5.86
7 days	1.055 ± 0.035	16.25 ± 0.35	55.4 ± 18.8
10 days	1.169 ± 0.46	19.7 ± 1.83	30.85 ± 3.46

[a]As determined by the percentage of cells staining with an anti-β-gal antibody (Abcam #ab9361) in the cell population. Data are presented as Arithmetic Mean ± Stdev (n = 2 for abemaciclib treated cells, n = 6 for DMSO treated cells). A representative example is shown; the experiment was repeated on 2 separate occasions.

Supplementary Table 4: Potency of abemaciclib for induction of hypermethylation of histone H3 at lysine 9 (H3K9me3)

Cell line	EC50 (µM) H3K9me3 levels[a]
MCF7	0.34 ± 0.016 (n = 2)
EFM-19	0.02316 (n = 1)

[a]H3K9me3 was detected using an antibody against this modification (Abcam cat# ab8898). EC_{50} values were obtained from a 10-point dose response experiment and are presented as the Arithmetic Mean ± Stdev.

Supplementary Table 5: Profiles of metabolites in MCF7 cells after treatment with abemaciclib

Metabolites	2 µM abemaciclib (3 DT)	0.5 µM abemaciclib (3 DT)	DMSO (3 DT)
Aspartate	0.47 ± 0.13	0.72 ± 0.10	1 ± 0.12
Glutamate	1.10 ± 0.38	0.71 ± 0.06	1 ± 0.07
Citrate	1.07 ± 0.3	1.20 ± 0.27	1 ± 0.28
Isocitrate	0.81 ± 0.52	1.90 ± 0.65	1 ± 0.33
α-ketoglutarate	0.08 ± 0.02	0.13 ± 0.03	1 ± 0.07
Succinate	0.65 ± 0.23	0.35 ± 0.08	1 ± 0.12
Fumarate	0.12 ± 0.07	0.12 ± 0.02	1 ± 0.13
Malate	0.09 ± 0.03	0.22 ± 0.01	1 ± 0.12
Lactate	0.38 ± 0.15	0.30 ± 0.02	1 ± 0.15
Pyruvate	3.59 ± 1.65	1.22 ± 0.64	1 ± 0.15
EC_{50} (µM) α-ketoglutarate	0.37 (1 DT)	0.28 (2 DT)	0.73 (3DT)

Levels of metabolites in MCF7 cells treated with abemaciclib or DMSO for 3 DT are shown; data are presented as the Arithmetic Mean + Stdev (n = 3 for abemaciclib). The EC_{50} values for the effect of abemaciclib treatment on α-ketoglutarate in MCF7 cells was obtained from a 10-point curve and corresponds to a single experiment at each treatment time.

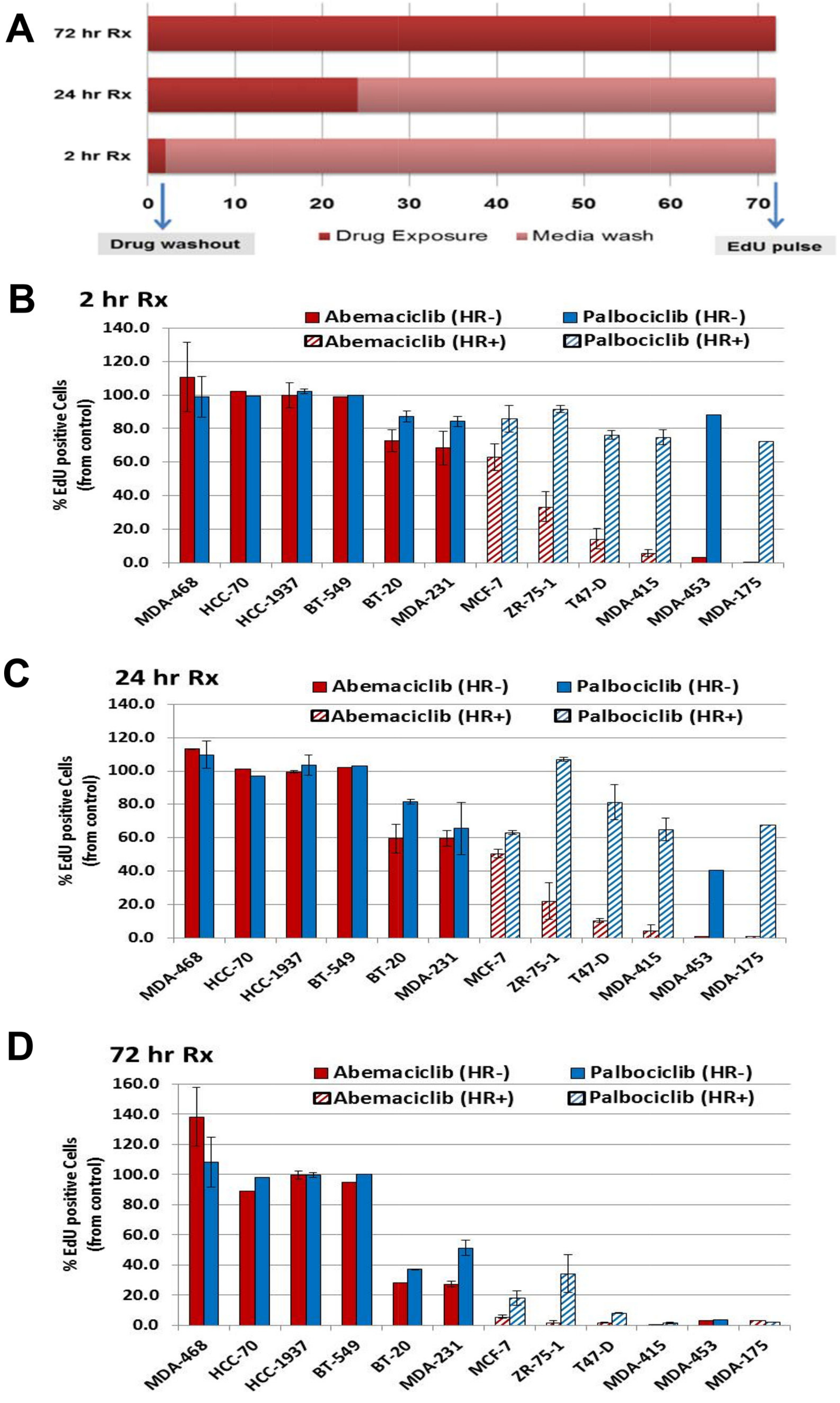

Supplementary Figure 1: Proliferative capacity of breast cancer lines after treatment with 500 nM abemaciclib or palbociclib. (**A**) Schematic of cell treatment and washout schedule. (**B**–**D**) Exposure times of 2 hr (B), 24 hr (C) or 72 hrs (D) with abemaciclib or palbociclib followed by drug washout as indicated in (A).

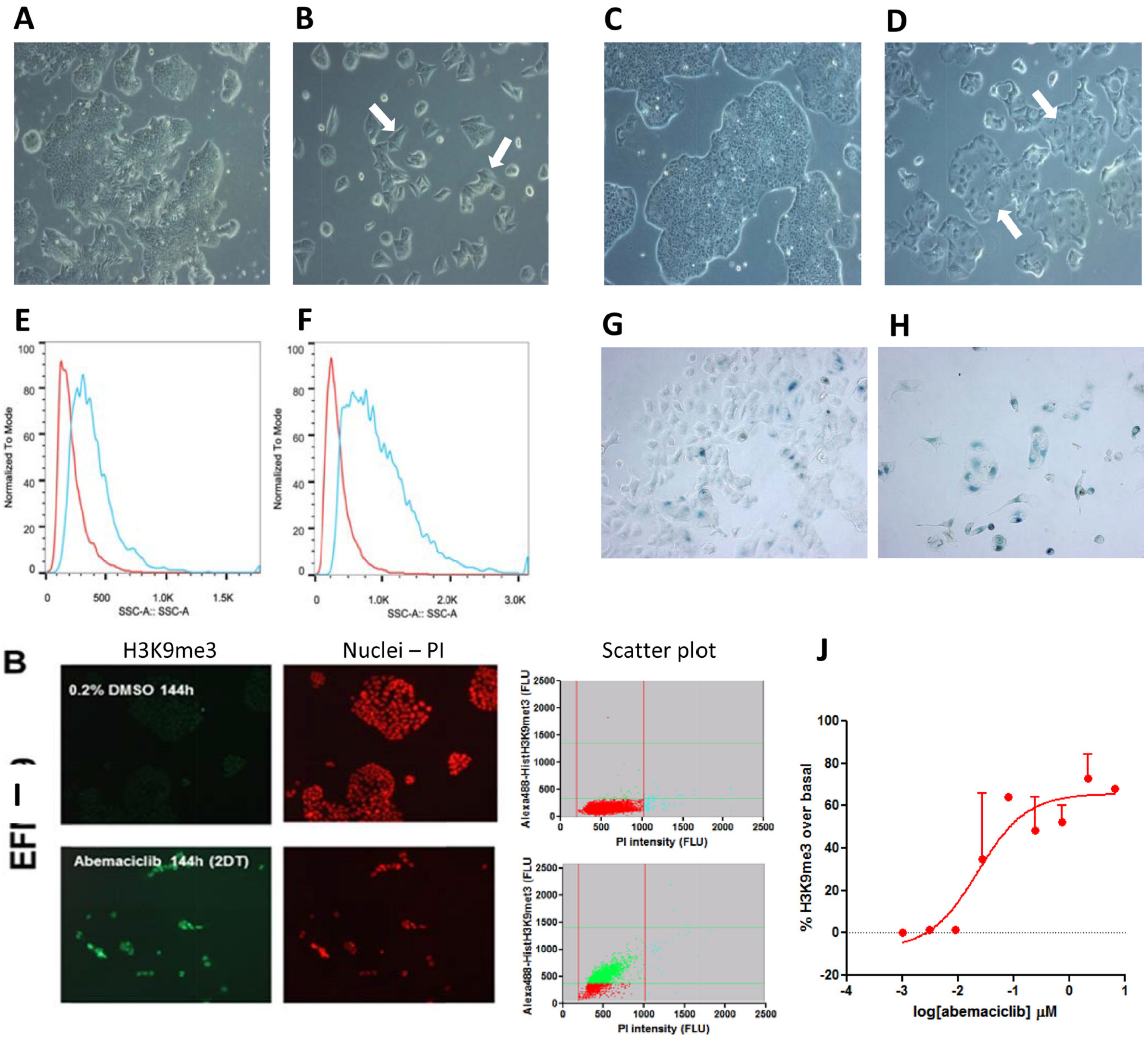

Supplementary Figure 2: ER+ breast cancer cell lines treated with abemaciclib show morphological changes, increased β-gal expression, and chromatin changes consistent with senescence. (**A**–**D**) Cells were treated with 0.2% DMSO (A MDA-MB-361, C EFM-19) or 0.5 μM abemaciclib (B MDA-MB-361, D EFM-19) for 3DT and images were taken at 100x by bright field microscopy (Leica DMiL). White arrows indicate evident morphological changes: enlarged and flatten cytoplasm upon treatment with abemaciclib. MDA-MB-361 (**E**) or EFM-19 (**F**) cells were treated with either DMSO (red line) or 0.5 μM of abemaciclib (blue line) for 3 DT and side scatter (SSC) analysis was carried out using flow cytometry and analyzed with FlowJo X software. (**G**, **H**) β-galactosidase expression in MCF-7 cells treated with 0.2% DMSO (G) and upon treatment with abemaciclib at 0.5 μM (H) after 4 days. (**I**) EFM-19 cells were treated with 0.2% DMSO or 0.5μM of abemaciclib for 2DT and immunofluorescence was carried out with an H3K9me3 antibody and PI. Microscopy images are shown on the left and scatter plots of total and H3K9me3 positive cell populations are represented on the right (Red = nuclei; green = H3K9me3 positive cells). (**J**) EFM-19 cells were treated for 2 DT with different concentrations of abemaciclib. % H3K9me3 was obtained by normalizing versus a control population and expressed as the number of objects stained with H3K9me3 antibody; the mean (SD) of 2 independent experiments at each concentration is plotted.

Targets inhibited > 50 % compared to vehicle

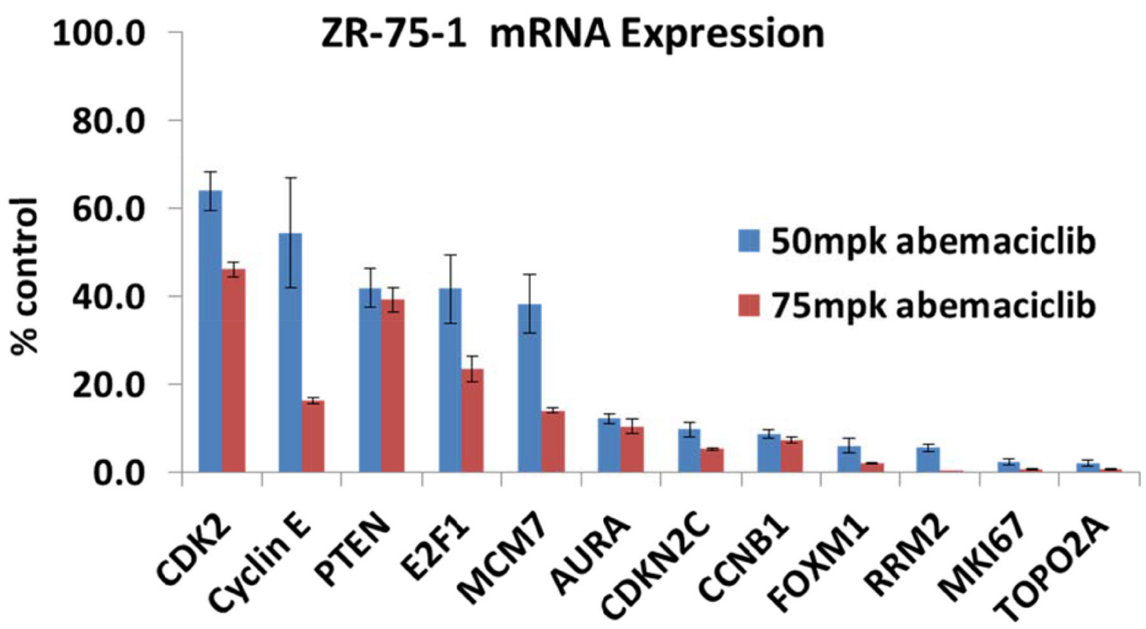

Supplementary Figure 3: NOD SCID mice (*n* = 5/group) implanted with ZR-75-1 xenograft tumors were treated by oral gavage (PO) once-daily for 5 days (QDx5) with vehicle or 50 or 75 mg/kg (mpk) of abemaciclib mesylate beginning on day 27 after tumor implantation. The xenograft tumors were collected 24 hours later and RNA was isolated. Each of the tumor samples was analyzed for the expression of 11 cell cycle regulatory genes which are displayed along the X-axis. The mean mRNA expression levels for each gene are represented as relative values (± SEM) as compared to the control tumors whereby the mean expression for each mRNA in the control tumors was given a value equal to 100%. The suppression of expression is observed in all situations wherein the relative expression is less than 100%. The genes evaluated in this assay were CDK2, Cyclin E, MCM7, CDKN2C (p18), E2F1, PTEN, Aurora A (AURA), cyclin B1 (CCNB1), FOXM1, ribonucleotide reductase subunit M2 (RRM2), Ki67 (MKI67) and Topoisomerase II alpha (TOPO2A).

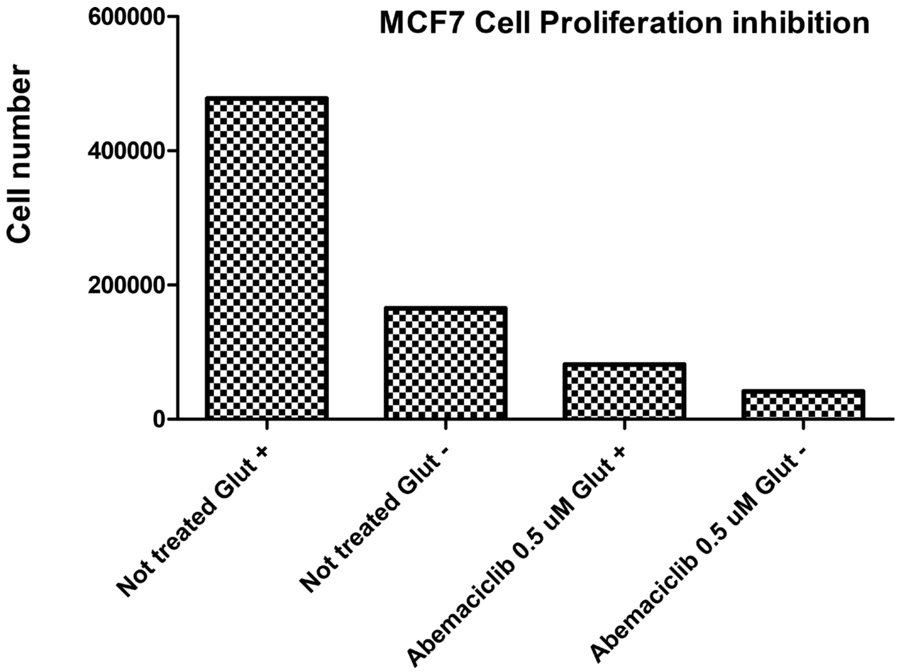

Supplementary Figure 4: Quantitation of cells shown on Figure 6A/D. Cells were collected after 2.5DT incubation and quantitated using flow cytometry after nuclei staining using propidium iodide. Data analysis was carried out using FlowJo® software.
